# Exercise and Dietary-Mediated Reductions in Postprandial Lipemia

**DOI:** 10.1155/2014/902065

**Published:** 2014-06-29

**Authors:** Eric P. Plaisance, Gordon Fisher

**Affiliations:** Department of Human Studies, Exercise and Nutritional Physiology Laboratory, University of Alabama at Birmingham, Birmingham, AL 35294, USA

## Abstract

Postprandial hyperlipemia produces long-term derangements in lipid/lipoprotein metabolism, vascular endothelial dysfunction, hypercoagulability, and sympathetic hyperactivity which are strongly linked to atherogenesis. The purpose of this review is to (1) provide a qualitative analysis of the available literature examining the dysregulation of postprandial lipid metabolism in the presence of obesity, (2) inspect the role of adiposity distribution and sex on postprandial lipid metabolism, and (3) examine the role of energy deficit (exercise- and/or energy restriction-mediated), isoenergetic low-carbohydrate diets, and omega-3 (n-3) fatty acid supplementation on postprandial lipid metabolism. We conclude from the literature that central adiposity primarily accounts for sex-related differences in postprandial lipemia and that aerobic exercise attenuates this response in obese or lean men and women to a similar extent through potentially unique mechanisms. In contrast, energy restriction produces only mild reductions in postprandial lipemia suggesting that exercise may be superior to energy restriction alone as a strategy for lowering postprandial lipemia. However, isoenergetic very low-carbohydrate diets and n-3 fatty acid supplementation reduce postprandial lipemia indicating that macronutrient manipulations reduce postprandial lipemia in the absence of energy restriction. Therefore, interactions between exercise/energy restriction and alterations in macronutrient content remain top priorities for the field to identify optimal behavioral treatments to reduce postprandial lipemia.

## 1. Introduction

Reductions in vocational physical activity and the availability and consumption of energy-dense foods are often cited as primary culprits of the rising incidence of obesity observed throughout the world [1–6]. The increased incidence of obesity has presented both health-related and economic challenges which exceed $110 billion dollars per year in the US alone [7], a level which surpasses any other country in the world. One of the conventional consequences of obesity (especially centrally distributed adiposity) is an increase in triglyceride (TG)-rich lipoproteins (TRLs) and associated hypertriglyceridemia. TRLs (very low-density lipoprotein (VLDL) and chylomicrons (CM)) reduce cholesterol content of high-density lipoproteins (HDL) and decrease the size of low-density lipoproteins (LDL) which increase the propensity for vascular endothelial infiltration and oxidation [8]. Fasting HDL-C concentrations often account for a greater amount of variance in the risk of cardiovascular disease (CVD) compared to TGs leading to their dismissal as a primary risk factor for atherosclerosis [9–11].

While the contribution of fasting TGs and HDL-C to the overall risk of CVD remains controversial [12, 13], accumulating evidence suggests that exaggerated postprandial lipemia produces rapid derangements of lipid/lipoprotein metabolism, vascular endothelial dysfunction, hypercoagulability, and sympathetic hyperactivity that is strongly linked to atherogenesis [10, 14–20]. A randomized clinical trial of 602 men and women illustrated that asymptomatic carotid atherosclerosis was positively correlated with postprandial lipemia [21]. Despite comparable fasting lipids, male offspring of men with CVD had significantly higher serum TGs up to 12 hours following a high-fat meal, indicative of a delayed clearance of TGs [22]. Furthermore, postprandial hyperlipemia increased the number of myocardial infarctions by 40% for every 100 mg/dL increase in Physician's Health Study and Multiple Risk Factor Intervention Trials [17, 23].

The relationship between postprandial lipid metabolism and atherosclerosis is not surprising when one considers that the majority of individuals following a typical Western Diet consume 3 to 5 meals per day. Since each meal requires 6–12 hours to fully dissipate TGs in circulation, the implication is that most individuals spend over two-thirds of the day in a postprandial state with elevated TRLs (postprandial hyperlipidemia) [24, 25]. The capacity to regulate incoming chylomicrons from exogenous sources, tasked with counterregulation of the endogenous production and secretion of VLDL-TG, may offer a more valid investigation of the capacity of tissues to appropriately regulate lipid metabolism. Therefore, interventions which improve the capacity to regulate tissue and blood lipid metabolism following a meal would be expected to lower CVD risk.

The purpose of the current review is to (1) provide a qualitative analysis of the available literature examining the dysregulation of postprandial lipid metabolism in the presence of obesity, (2) inspect the role of adiposity distribution and sex on postprandial lipid metabolism, and (3) examine the role and mechanisms by which energy deficit produced via exercise and/or energy restriction, isoenergetic low-carbohydrate diets, and n-3 fatty acid supplementation improves postprandial lipid metabolism.

## 2. Obesity and Postprandial Lipemia

Systematic abnormalities in fasting and postprandial blood lipid and lipoprotein metabolism have been well documented in obesity and other conditions which produce hyperinsulinemia/insulin resistance [26]. Insulin resistance produces de novo lipogenesis, increases microsomal triglyceride transfer protein, and enhances intracellular apolipoprotein B48 stability in the intestine [27, 28]. Along with increased free fatty acid loading, increases in intestinal de novo lipogenesis increase the secretion of apolipoprotein B48 from enterocytes which increases the absorption and circulating concentrations of chylomicrons [28]. The increase in secretion is accompanied by a reduction in the fractional catabolic rate of apolipoprotein B48. Reductions in adipose tissue lipoprotein lipase (LPL) mass and activity [26, 29, 30] have also been observed in the presence of obesity-mediated insulin resistance. Furthermore, downregulation of LDL-receptor expression [31] leads to reductions in remnant lipolysis and removal [28]. Concurrently, obesity associated hepatic insulin resistance dampens the repression of VLDL-TG secretion normally observed in the presence of hepatic insulin sensitivity. The additional VLDL-TG competes with chylomicrons for hydrolysis by LPL and hepatic receptors, thereby reducing the overall clearance of serum TGs [28, 29].

### 2.1. Adiposity Distribution and Sex

Subsequent studies have attempted to address the precise role of adiposity distribution on postprandial lipemia. Overall, visceral adipose tissue distribution appears to be a better predictor of postprandial TG responses than body mass index (BMI) alone or gynoid distribution of adipose tissue [32–35]. Since the distribution and magnitude of visceral adiposity is greater in men than in women, some have proposed that these differences may be responsible for gender dimorphisms in postprandial lipemia and rates of CVD [34, 36].

In agreement with this interpretation, variation in postprandial TG responses between men and women was abrogated after controlling for visceral adiposity suggesting that the distribution of body fat is responsible for postprandial TG responses as opposed to sex differences [34, 37]. Furthermore, Mekki et al. [35] showed that android obese women had greater postprandial lipemia than women who were lean or exhibited a gynoid distribution of excess adiposity despite the presence of comparable BMI and fasting TGs. In addition, postprandial lipemia was similar between normolipidemic lean and gynoid obese groups, suggesting that excess adipose tissue distribution away from the viscera is insufficient to alter postprandial lipid metabolism. When men with impaired or normal glucose tolerance were individually matched for visceral adipose tissue accumulation, no significant differences were found in postprandial responses of all TRL-TG fractions between groups [38]. Additional studies [39, 40] showed similar results suggesting that visceral adipose tissue accumulation may be a more important determinant of postprandial lipemia than impaired glucose tolerance. These findings suggest that insulin resistance/hyperinsulinemia-mediated increases in visceral adiposity may be responsible for postprandial hyperlipemia. Future studies are needed to address cause and effect between visceral adiposity and insulin resistance* per se*. Taken together, abnormal postprandial lipemia can be detected in the presence of excess visceral adiposity with comparable BMI and in the absence of fasting hypertriglyceridemia, providing further support that postprandial lipemia is a more sensitive predictor of metabolic and CVD risk than fasting parameters.

## 3. Exercise and Postprandial Lipemia

### 3.1. Exercise Training Produces Effects on Postprandial Lipemia That Are Primarily Associated with Energy Expended during the Most Recent Bout of Exercise Performed

Cross-sectional and longitudinal studies show that regular aerobic exercise reduces postprandial lipemia in the presence or absence of weight loss [41–51]. However, the effects of aerobic exercise training on postprandial lipemia are diminished when blood sampling occurs 48 hours after the most recent bout of exercise suggesting that exercise produces acute effects that are rapidly reversed [49, 50]. Detraining studies provide further support for the concept that exercise performed in the hours prior to a mixed or high-fat meal is responsible for generating a metabolic environment which leads to postprandial lowering of TGs. Indeed, endurance-trained individuals who discontinued training for more than 60 hours had postprandial circulating TGs that were over 35% higher than levels following the last exercise training session [51]. Direct evidence for this phenomenon has been observed in multiple populations following single sessions of exercise to provide unequivocal evidence that exercise training produces important acute metabolic responses that reduce postprandial lipemia.

### 3.2. Timing and Composition of Test Meals

Although the nutrient composition of test meals, populations studied, and exercise conditions/modalities employed are highly variable throughout the literature, several conclusions can be drawn that support the therapeutic benefits of consistent exercise participation as a means to improve metabolic health in the postprandial state. For instance, significant disparities exist between studies regarding the type of test meals employed to evaluate the impact of exercise on postprandial lipemia. The literature is split regarding the use of mixed high-fat meals and exclusively high-fat meals to explore these effects. Although the validity of high-fat diets exclusively as a test meal has come into question, what is important is that, in the majority of cases, a single session of aerobic exercise robustly lowers postprandial lipemia regardless of the composition of the test meal.

It is important to point out that the amount of dietary fat in the test meal required to increase plasma TG concentrations appears to be dose-dependent [22]. Absolute doses of 5–15 g do not increase postprandial lipemia (29–32); whereas doses of 30–50 g increase postprandial lipemia by 75 to 110 mg/dL above baseline. Doses above 80 g exaggerate postprandial lipemia but are similar in magnitude to the increase with 50 g of dietary TG suggesting that a threshold is achieved beyond a certain dose of TGs consumed.

Another important aspect regarding test meal design and administration that should be considered is the fact that although the single test meal provides a powerful laboratory design, it may not reflect a real-world situation that occurs as additional meals are consumed. Farah and colleagues [52] addressed the question of how subsequent meals impact the capacity of exercise to lower postprandial lipemia and reported that prior exercise lowered the overall TG response to 3 meals. These results indicate that aerobic exercise reduces the postprandial TG response to multiple meals suggesting that the observed effects are not limited to effects on a single meal or the laboratory setting.

### 3.3. Timing of Exercise Interventions

The majority of investigations which have examined the effects of exercise on postprandial lipemia have been designed where a single session of aerobic exercise is conducted 11–20 hours prior to the test meal in a 2-day design. On day 1, participants perform a prescribed exercise modality and on day 2 they receive a test meal followed by temporal blood sampling for up to 8 hours [16, 53–64]. In contrast, others have used exercise sessions conducted 30–60 minutes before [65–67], 4 hours before [68], or 60–90 minutes after a meal [65, 66] to examine the effects of a single bout of exercise on postprandial lipemia. Aerobic exercise conducted 11–20 hours before the test meal appears to produce the greatest magnitude of reduction in postprandial lipemia compared to aerobic exercise conducted 30 minutes to 4 hours before a test meal [65, 66]. Finally, aerobic exercise conducted 1 hour following a high-fat meal produced only modest reductions in postprandial lipemia suggesting that prior exercise is superior to exercise conducted after a meal [65].

### 3.4. Effects of Exercise on Postprandial Lipemia Are Dose- and Intensity-Related

Early studies demonstrate that total energy expenditure achieved through isoenergetic low- or moderate-intensity aerobic exercise produces similar reductions in postprandial lipemia [54, 55], with exceptions [69]. Tsetsonis [54] reported that normolipidemic young men and women had lower postprandial lipemia after walking on a treadmill for 90 minutes at 61% of VO_2max⁡_ and that 90 minutes of walking at 31% of VO_2max⁡_ elicited little effect providing initial evidence that the effects of aerobic exercise on postprandial lipemia were due to either greater intensity or to greater energy expenditure at a higher intensity. In an attempt to answer this question, the authors conducted a follow-up study where they compared the effects of isoenergetic low- and moderate-intensity walking on postprandial lipemia [55]. The authors reported that 3 hours of walking at low-intensity (32% VO_2max⁡_) or walking for 1.5 hours at moderate-intensity (63%) (~1000 kcal energy expenditure for both) decreased postprandial lipemia to a similar extent. Further evidence that the beneficial effects of aerobic exercise on postprandial lipemia are related to the magnitude of energy expended come from studies which show that the accumulation of aerobic exercise throughout the day in multiple bouts produces similar benefits as a single session of isoenergetic aerobic exercise conducted at the same intensity [56, 58, 61–63, 70–74]. In contrast, a single session of aerobic exercise at 65% VO_2max⁡_ designed to expend ~1100 kcals reduced postprandial TG area under the curve (AUC) by 39% whereas isoenergetic exercise at 25% VO_2max⁡_ produced a statistically insignificant 9% decrease [69]. A recent study by Kim and colleagues [75] showed that isoenergetic low (25% VO_2max⁡_) and moderate (65% VO_2max⁡_) intensity aerobic exercise both lowered the TG AUC_I_, but moderate intensity was over 17% more effective than low-intensity exercise. Available evidence suggests that energy deficit may be the most important determinant of the magnitude of reduction in postprandial lipemia with exercise. However, multiple investigations suggest that single or accumulated bouts of moderate-intensity exercise lower postprandial lipemia to a greater extent than isoenergetic exercise of low intensity [61, 69, 75].

### 3.5. High-Intensity Interval Exercise

Although total energy expenditure achieved during and after an exercise bout may outweigh the role of intensity in low- to moderate-intensity aerobic exercise, an interesting observation from emerging studies is that high-intensity, short-duration interval exercise may yield results which are at least as effective as low- to moderate-intensity exercise of longer duration despite lower energy expenditure [76]. High intensity interval (HII) exercise consists of multiple short-term (30 s–240 s) bouts at >85% VO_2max⁡_ followed by 60 s–180 s of active or passive recovery periods. Indeed, moderate-intensity walking for 30 minutes at an energy expenditure of 240 kcals produced no effect on postprandial lipemia whereas HII exercise expending 103 kcals produced reductions in the TG AUC [76]. Trombold et al. [77] found that moderate-intensity and HII exercise both decreased the AUC_I_, but reported that HII exercise was more effective than moderate-intensity exercise for lowering postprandial lipemia despite identical energy expenditure during the exercise session. In contrast, isoenergetic (500 kcal) moderate-intensity and HII exercise decreased TG concentrations to a similar extent [73]. These differences may be due to disparities in the timing of test-meals as the meals were delivered 30 minutes after exercise in the latter study [73] versus 12–16 hours in the former study [77]. Additional studies show that sprint interval cycling produced no effects on postprandial lipemia [78, 79]. While additional studies are needed to sort out the role of HII exercise on postprandial lipemia, the notion that prior HII exercise can lower postprandial lipemia to a similar extent as moderate-intensity exercise, despite lower energy expenditure and time commitment, has important practical implications for individuals with limited access to exercise opportunities.

### 3.6. Is Resistance Training an Effective Modality to Reduce Postprandial Lipemia?

A growing number of investigations have examined the effects of resistance training on postprandial lipemia and its efficacy in comparison to traditional aerobic exercise training. Available evidence suggests that resistance exercise is an effective strategy to reduce postprandial lipemia [80–84], although exceptions exist [85–87]. In fact, Burns and colleagues [87] showed that resistance training actually increased postprandial lipemia following a test meal. Studies which show a reduction in postprandial lipemia with resistance exercise observe these changes in recreationally active normal weight, normolipidemic males and females [80–82] and sedentary obese females [83]. Parallel comparisons of aerobic and resistance exercise have been somewhat disappointing on the surface in the sense that each of the studies conducted showed that aerobic exercise produced no effect on postprandial lipemia (which is inconsistent with the aerobic exercise literature). Some have suggested that although energy expenditure was similar in magnitude to levels which have previously shown reductions, it is possible that an intensity threshold was not met (~30% VO_2peak_). However, it is important to note that reductions in postprandial lipemia were observed at a similar energy expenditure as that conducted for aerobic exercise suggesting that resistance training may be more effective than aerobic exercise at lowering postprandial lipemia. Support for this comes from a recent study in obese premenopausal women where the authors had the same group of women complete 2 separate 60 minute trials (aerobic exercise at 60–65% VO_2peak_ and resistance training exercise) [83]. Although they did not measure energy expenditure, it would be predicted that energy expenditure in the resistance training condition was approximately 50% lower than the aerobic exercise group. Taken together, these results suggest that resistance training exercises that work the upper and lower body may be more efficacious than aerobic exercise at lowering postprandial lipemia.

Overall, the literature suggests that the energy expenditure requirements to produce reductions in postprandial lipemia occur at a minimum of 250 kcals, but occur more consistently at 400 kcals and greater during low- to moderate-intensity aerobic exercise [88]. Zhang et al. [59] showed that 400 kcals produced similar results to that of EE over 700 kcals suggesting that an upper capacity may be achieved where only limited returns are produced beyond a certain level of energy expenditure. Future studies will be required to examine the effects of HII exercise (>85%) and resistance training to provide additional evidence that energy expenditure requirements are different with higher intensity exercise.

### 3.7. What Are the Mechanisms Responsible for Exercise-Mediated Reductions in Postprandial Lipemia?

**Figure 1:**
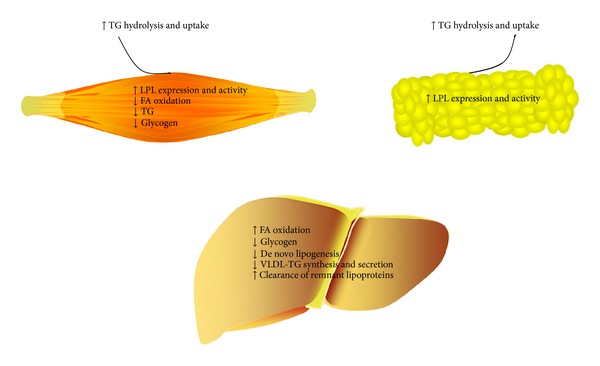
Potential mechanisms by which prior exercise reduces postprandial lipemia. Aerobic exercise has been shown to increase FA substrate utilization during metabolic studies presumably in skeletal muscle and liver. Increased hepatic FA oxidation and decreased de novo lipogenesis are thought to decrease VLDL-TG synthesis and secretion. Increased clearance of postprandial triglycerides through upregulation of LPL expression and activity has also been proposed in skeletal muscle and to a lesser extent in adipose tissue.

Prior exercise reduces postprandial lipemia by three possible mechanisms acting alone or in combination: (1) decreased appearance of chylomicron-TG concentrations from the gut, (2) increased clearance of TRLs (VLDL and/or CM) via exercise-mediated increases in skeletal muscle and/or adipose tissue LPL activity, and (3) decreased hepatic VLDL-TG secretion [89] (Figure 1). Although studies are limited, available evidence indicates that aerobic exercise does not reduce gastric emptying, as determined by paracetamol administration [89–91] and does not delay the time to achieve peak TG concentrations compared to control conditions [89, 91, 92] which would be expected to occur if exercise altered the rate of gastric emptying.

In contrast, numerous investigations have reported that exercise produces a delayed and transient increase in LPL protein expression and activity that occurs within 4 to 8 hours after exercise and remains elevated for up to 24 hours [93–100]. These results are metabolically relevant as skeletal muscle LPL activity increases the hydrolysis of TG and clearance of free fatty acids in the postabsorptive state when serum insulin concentrations are relatively low. Therefore, an increase in skeletal muscle LPL activity produced by exercise would be expected to increase the clearance of circulating TG in the postabsorptive state. There is a strong association between the elevation in LPL activity and reductions in fasting serum TGs in most [96, 97, 100–102] but not all investigations [97, 103, 104].

Studies which have examined the effects of aerobic exercise on postprandial lipemia when exercise was performed 12–18 hours before a meal consistently show significant reductions in TGs. However, the effects of exercise on LPL and TG clearance are unclear. For example, 90 minutes of walking reduced postprandial lipemia in men, but the same amount of exercise did not increase clearance of an intravenous lipid emulsion [90]. Herd and colleagues [105] showed that 90 minutes of moderate-intensity aerobic exercise conducted 18 h before a meal reduced postprandial lipemia but did not produce statistically significant increases in LPL activity (although the individuals who experienced increases in LPL activity after exercise had the greatest reduction in TGs). An additional study showed that 2 hours of moderate-intensity exercise did not significantly increase TG clearance across the leg 18 hours after exercise despite lowering postprandial TG concentrations [92].

While a role for skeletal muscle LPL in the reduction of postprandial lipemia cannot be ruled out, multiple factors suggest that other mechanisms must be involved. For example, it is well known that postprandial hyperinsulinemia promotes the storage of TG in adipose tissue by increasing LPL activity [106, 107] and that skeletal muscle LPL activity and TG storage are minimal [105, 108, 109]. The question is whether exercise-mediated increases in skeletal muscle LPL activity are maintained in the presence of hyperinsulinemia as in the postabsorptive state or if exercise increases adipose tissue LPL activity. Furthermore, the increase in skeletal muscle LPL activity is likely to have dissipated in most protocols where exercise is conducted the day before the test meal.

Early studies in rodents revealed that aerobic exercise training reduced the secretion of hepatic VLDL-TG by decreasing serum NEFAs and de novo lipogenesis [110–112]. While direct evidence in humans is limited, emerging studies indicate that reductions in serum TG produced by aerobic and resistance exercise are attenuated by reductions in either hepatic VLDL-TG secretion or clearance. Indeed, 50–70% of the reduction in postprandial lipemia produced by aerobic exercise was accounted for by reductions in hepatic VLDL-TG secretion [105, 113, 114]. In one of the most elegant investigations conducted to date, Davitt and colleagues [83] provided evidence using stable isotopes that the reduction in postprandial lipemia with aerobic and resistance training was not achieved by enhanced clearance of dietary fat, but instead by reduced abundance of endogenous fatty acids in circulating TGs. Others [115] reported that a single session of aerobic exercise reduced postprandial lipemia by decreasing hepatic secretion and increasing clearance in women whereas in men, the reduction in postprandial lipemia was due to increased clearance alone. From the available literature, it is difficult to draw strong conclusions about the specific roles and magnitude of reduction in postprandial lipemia produced by hepatic TG secretion and clearance. Nonetheless, it seems plausible that reductions in VLDL-TG secretion may be the primary mechanism by which exercise reduces postprandial lipemia. A role for postprandial TG clearance may exist, especially with HII exercise. For example, beta-hydroxybutyrate (BOHB) concentrations were not different from control following a single HII exercise bout [76] suggesting that the reduction in TG was not due to reductions in hepatic production of VLDL. While this conclusion should be reached with caution due to the equivocal role of BOHB as a valid marker of VLDL production [116, 117], it does suggest that reductions in postprandial lipemia may be attributed, at least in part, to increased clearance. It is possible that HII exercise may more effectively alter LPL mass and activity than low- to moderate-intensity exercise.

The mechanisms by which aerobic and resistance exercise decrease VLDL-TG secretion and postprandial lipemia is unclear. We propose the possibility that aerobic exercise-mediated reductions in hepatic and skeletal muscle glycogen content is part of a metabolic program produced by exercise which preferentially shuttles fatty acids to oxidation as a strategy to spare glucose for storage and to reestablish intracellular ATP concentrations, thereby lowering the packaging/secretion of VLDL-TG and potentially increasing clearance. Evidence for this hypothesis is supported by the finding that a prior bout of endurance or resistance exercise increases whole body fatty acid oxidation [80, 91, 118, 119] and increases glycogen synthase activity and protein expression in skeletal muscle [120]. This hypothesis is also supported by the findings of some studies which show that moderate-intensity exercise produces greater reductions in postprandial lipemia than low-intensity exercise (where carbohydrate oxidation would presumably be lower than moderate intensity exercise) [61, 69, 75]. In contrast, if moderate-intensity exercise produces similar reductions in postprandial lipemia as isoenergetic low-intensity exercise as previously reported [55] this would suggest an alternative possibility. This possibility is further suggested by the observation that aerobic exercise produced similar reductions in postprandial lipemia in the presence or absence of pharmacological inhibition of adipose tissue lipolysis by acipimox [121]. If the increase in glycogen oxidation is correct, then it would be expected that exercise in the presence of acipimox would produce a greater reduction in postprandial lipemia, but this was not the case. Overall, these results suggest that regardless of the substrate used, the hepatic and skeletal muscle program following exercise is to preferentially use fatty acids from TRLs to replenish ATP thus sparing glucose for glycogen storage as part of the training adaptation for subsequent exercise bouts. This is in agreement with studies which show that exercise increases glycogen synthase activity and protein expression in skeletal muscle [120].

### 3.8. Does Acute Energy Deficit Produced by Diet Yield Similar Effects on Postprandial Lipemia as Energy Deficit Produced by Exercise?

An important question arising from acute exercise studies is whether the accompanying energy deficit is responsible for the reduction in postprandial lipemia. To address this question, Gill and Hardman [16] compared an energy deficit of 500 kcals produced by exercise with an equivalent deficit produced by energy restriction. Exercise decreased total and incremental TG concentrations while decreasing energy intake an equivalent amount produced only mild reductions in serum TG concentrations that were 3-fold lower than that produced by exercise. In a similar fashion, Maraki and colleagues [122] reported that isoenergetic deficits created by energy restriction or aerobic exercise decreased postprandial lipemia to a similar extent statistically, with a numerically stronger effect elicited by exercise compared to energy restriction. The interpretation of the results is complicated by the fact that Gill and Hardman [16] reported that the energy deficit caused by energy restriction was 17% lower than that induced by exercise. Based on limited evidence, it appears that greater amounts of energy restriction are required to produce reductions in postprandial lipemia compared to the energy deficit created by exercise.

Using a different approach, Burton et al. [123] showed that moderate-intensity aerobic exercise producing an energy deficit of ~668 kcal lowered postprandial lipemia as expected. However, when an isoenergetic mixed meal was provided shortly after exercise to achieve energy balance, the effects of aerobic exercise on postprandial lipemia were dramatically attenuated. Additional evidence in men using a combination of moderate- and high-intensity exercise showed that aerobic exercise produced a profound reduction in postprandial lipemia which was ameliorated when glucose was consumed shortly after exercise to reestablish energy balance [124].

The results of these studies suggest that dietary and exercise-induced reductions in postprandial lipemia may be mediated through both common and different pathways [16, 123]. These differences may be related to the fact that moderate-intensity exercise produces quantitatively larger deficits in skeletal muscle and liver glycogen than energy restriction alone [123]. As described above, the reduction in glycogen content in both tissues would be expected to preferentially partition intracellular glucose to storage as glycogen and increase the hydrolysis and uptake of circulating TG to provide fatty acids as a substrate for oxidation. In contrast, energy restriction-mediated deficits would likely use a greater proportion of adipose tissue TGs which would not generate an equivalent reduction in skeletal muscle and liver glycogen content. While the energy deficit created by exercise appears to be a primary mediator of the exercise-induced reduction in postprandial lipemia, energy balance created by carbohydrate replacement alone abolished the reduction in postprandial lipemia by exercise providing further support that glycogen resynthesis and energy balance are driving forces in this process. Additional studies are required to further evaluate the role of carbohydrate and other dietary macronutrients such as protein or fat to determine the interaction between macronutrient replacement, energy balance, and exercise-mediated reductions in postprandial lipemia.

## 4. Energy and Carbohydrate Restriction Effects on Postprandial Lipemia

Accumulating evidence indicates that negative energy balance, achieved through increases in energy expenditure or decreases in energy intake, decrease postprandial lipemia [89, 125]. While studies to date have focused on assessing the absolute energy deficit on postprandial lipemia, it has been suggested that the improvements from either diet or exercise may be greater when there is a larger carbohydrate deficit [124]. Indeed, this is supported by dietary studies that demonstrate greater improvements in fasting serum lipids and significantly greater reductions in postprandial lipemia following a very low-carbohydrate diet as compared to a low-fat diet, and exercise studies that have shown that acute improvements in postprandial TG metabolism is abolished if a carbohydrate rich postexercise meal is consumed [124, 126]. Thus, it is possible that a low-carbohydrate diet may be more beneficial than a low-fat diet for improving both fasting plasma lipids and postprandial lipemia.

A reduction in the intake of dietary fat has long been recommended as a means to reduce risk factors associated with metabolic and CVD [127]. However, it is generally thought that a significant amount of weight loss must occur to appreciate the beneficial effects of a low-fat diet. Furthermore, it has been shown that low-fat diets lower HDL-C concentrations and increase fasting plasma TG concentrations [128, 129], which is associated with an increased risk of development and mortality from CVD [130]. While it has been suggested that hypertriglyceridemia is a relatively short-term adaptive response to the increase in dietary carbohydrate [131], results from epidemiological studies show that this response may actually be a longer lasting phenomenon [132]. Thus, it remains to be determined whether or not carbohydrate-induced hypertriglyceridemia is transient in nature or can be avoided. An overview of the literature suggests that the carbohydrate-induced hypertriglyceridemic response can only be partially normalized if there is a significant (at least 10%) amount of weight loss that accompanies the low-fat high-carbohydrate diet [133]. Given these observations, more recent studies have begun to focus on the potential benefit of reducing carbohydrate content in the diet as a means to improve fasting and postprandial lipid metabolism [125, 126, 129, 134].

Carbohydrate restriction has been adopted by a large number of people; however, because carbohydrate restriction can increase the production of ketone bodies, there has been caution issued by the scientific community in regard to the safety of these diets. This concern has prompted the USDA to call for further research into the safety and efficacy of low-carbohydrate diets. Thus, in recent years a number of well-designed studies have tested the effects and safety of a low-carbohydrate diet on reducing biomarkers for CVD risk, and improving fasting and postprandial lipid metabolism. There have been several well-controlled randomized studies comparing the effects of a carbohydrate-restricted diet with a fat-restricted diet on weight loss, blood lipids, and other CVD risk markers. Given that responses may differ between genders, weight status, and blood lipid profile, there have been several investigations assessing a number of different cohorts, including men and women with atherogenic dyslipidemia [135], normal weight-normolipidemic men [136, 137], normal weight-normolipidemic women [126], and overweight men [125].

In a cohort of normal weight men [136] and women [126] assigned to an isoenergetic diet comprised of a low-carbohydrate diet or low-fat diet, there was a reduction in fasting TG, postprandial lipemia, and fasting insulin compared to low-fat diet in men and a significant increase in postprandial lipemia compared to low-fat diet in women. However, in a cohort of overweight men that incorporated a hypocaloric diet, both a low-carbohydrate and a low-fat diet led to a reduction in total cholesterol, fasting insulin, and HOMA-IR. In contrast, fasting TAG, fasting glucose, and increases in mean and peak LDL particle size were only reduced by the low-carbohydrate diet [125]. Overall, the authors concluded that the hypocaloric low-carbohydrate diet had a similar or better effect on overall blood lipids compared to the low-fat diet. In a more recent study in men and women with MetS, Volek et al. [135] found that carbohydrate restriction led to a two-fold greater weight loss as compared to the low-fat control. Additionally, carbohydrate restriction resulted in a significantly greater reduction in fasting glucose and HOMA-IR, lower total postprandial TG AUC following an oral fat load, reduction in fasting TG and total cholesterol, and increased circulating HDL and LDL particle size as compared to a fat restricted diet [135]. Thus, carbohydrate restriction provided a more comprehensive improvement in clinical risk factors associated with MetS than fat restriction at a reduced caloric intake. In summary, it appears that a very low-carbohydrate diet may represent an alternative and safe strategy for metabolic and cardiovascular health that extends beyond weight regulation.

In addition to the theory that insulin resistance may exacerbate both fasting and PPL, there is also accumulating evidence that the type of sugar may differentially regulate these effects [138]. Fructose, in particular, has been given a great deal of attention due to its potential role for improving glucose tolerance and attenuating the postprandial insulin response [139, 140]. However, these responses have been shown in smaller doses of dietary fructose (7.5 g), whereas higher doses have been shown to increase postprandial lipemia [141]. Thus, while fructose may attenuate the postprandial glucose response it may potentiate postprandial hyperlipemia [138, 142]. While the exact mechanism in which fructose potentiates postprandial lipemia remains to be determined, current data in humans suggests a decrease in activation of adipose tissue LPL activity due to a lower postprandial insulin excursion [138]. It is also possible that fructose-mediated increases in hepatic de novo lipogenesis [143, 144] increase postprandial VLDL synthesis and secretion. Additional studies in rodents suggest a reduction in hepatic clearance of VLDL-TG [145]. The role of fructose consumption on postprandial lipemia is an area that warrants further investigation and may explain why high-carbohydrate diets can exaggerate postprandial lipemia in individuals that are insulin sensitive.

### 4.1. Potential Mechanisms in Which Carbohydrate Restriction Improves Fasting and Postprandial Lipemia

In order to discuss potential mechanisms in which carbohydrate restriction improves fasting and postprandial lipemia, it is important to first discuss potential ways in which carbohydrate consumption can induce lipemia and increase fasting TG. Simply put, an increase in the fasting TG and postprandial lipemia response is a result of either an increase in hepatic de novo lipogenesis or a reduction in skeletal muscle and/or adipose tissue TG clearance. Carbohydrate-induced lipemia was first recognized in the early 1950s, where low-fat diets were prescribed to lower blood cholesterol levels [146, 147]. Paradoxically, when investigators prescribed this diet, patients exhibited postprandial lipemia [146]. The proposed mechanisms for postprandial hyperlipemia in early studies of low-fat/higher carbohydrate diets were that higher carbohydrate promoted hepatic TG synthesis and a net decrease in TG removal rates leading to a more pronounced lipemia [148]. Shortly thereafter, it was recognized that the carbohydrate-induced hypertriglyceridemic response was exacerbated in individuals that were insulin resistant [149]. Reaven et al. [150] performed a number of studies demonstrating that even mild peripheral insulin resistance could result in failure of an inhibition of lipolysis when insulin is elevated, and also increases hepatic TG secretion due to a reduction of insulin's ability to inhibit liver TG secretion when hepatic insulin resistance was present [151]. Given these observations, peripheral insulin resistance has become one of the prevailing theories in which high-carbohydrate diets increase fasting and postprandial lipemia. Over the ensuing years, a number of mechanisms have been identified that may explain carbohydrate-induced lipemia, including increased secretion of hepatic VLDL particles and/or upregulation of apolipoprotein synthesis and TG packaging per VLDL particle, or a reduction in clearance due to a decrease in LPL activity [133]. Additionally, while the lipemic response to high carbohydrate has been clearly demonstrated, it is important to note that evidence exists that reveals lower lipemia when weight loss occurs or less fat composition is included in the diet [133]. Thus, hyperinsulinemia, hepatic and peripheral insulin resistance, and body weight all appear to be critical factors associated with the magnitude of carbohydrate-induced lipemia.

While the paradoxical postprandial lipemia response following a high-carbohydrate/low-fat diet was observed over 50 years ago, the idea of lowering carbohydrate and increasing fat composition in the diet has only begun to gain traction over the last 5–10 years. The precise mechanisms in which a low-carbohydrate diet improves postprandial lipemia remain to be determined. However, it is currently thought that reducing carbohydrate intake reduces fasting and postprandial TG by reducing VLDL production rate and increasing TG removal by increasing both plasma and skeletal muscle LPL activity [135, 152]. It has also been shown that carbohydrate restriction can decrease fasting and postprandial insulin concentrations [135]. This is important since insulin suppresses skeletal muscle and adipose tissue lipolysis and increases de novo lipogenesis. In the presence of insulin resistance, an increase in skeletal muscle lipolysis and a decrease in hepatic lipid storage would be expected, leading to increased production of larger TG-enriched LDL particles, an increased formation of small LDL particles, and a decrease in HDL-C. In addition to the effects of insulin on hepatic lipid production, it is also possible that there is greater lipid clearance due to a shift towards fat oxidation and a lower reliance on insulin for skeletal muscle glucose uptake when fat content is increased in the diet. The role of insulin resistance in determining lipemia has been shown by Petersen et al. [153]. Dietary carbohydrate substrate partitioning, liver and muscle TG and glycogen synthesis, and de novo lipogenesis were determined using ^1^H and ^13^C NMR spectroscopy and deuterium enrichment. They found that insulin resistant men had impaired skeletal muscle and hepatic glycogen formation following carbohydrate intake, directed dietary carbohydrate toward hepatic de novo lipogenesis, and TG synthesis and produced an overall increase in plasma TG concentrations [153]. Thus, it is likely that the benefits of carbohydrate restriction would be greater in individuals with insulin resistance. Indeed, it has been shown that carbohydrate restriction can decrease malonyl-CoA concentrations, removing the disinhibition of carnitine acetyltransferase and enabling greater fatty acid transport and fatty acid oxidation [152]. Furthermore, lowering carbohydrate intake for as few as three days has been shown to upregulate genes associated with fatty acid oxidation [154].

## 5. Omega-3-Fatty Acids

The omega-3 (n-3) fatty acids, docosahexaenoic acid (DHA), and eicosapentaenoic acid (EPA) beneficially modify fasting/postprandial blood lipid and lipoprotein metabolism and independently decrease mortality due to myocardial infarction and sudden death [155]. An analysis of 72 placebo-controlled human studies of at least 2 weeks in length providing 2 to 7 grams of n-3 fatty acids per day found that fish oils dose-dependently decrease serum fasting TG concentrations in normo- and hypertriglyceridemic individuals by 4% to 40% [155, 156].

The effects of n-3 fatty acids have also been explored to determine their impact on postprandial lipid/lipoprotein metabolism. Tinker and colleagues showed that a liquid fish oil supplement containing 5.2 g of EPA and DHA decreased postprandial TGs in hypertriglyceridemia participants [157]. Similar results have been observed by others in both normo- and hypertriglyceridemic participants [158–160] with exceptions [161, 162]. Although the precise mechanisms are unknown, fish oil supplementation has been shown to decrease hepatic triglyceride synthesis via inhibition of diacylglycerol transferase (DGAT), fatty acid synthase, and acetyl coenzyme A carboxylase ACC activities [163, 164]. Fish oils also enhance fatty acid oxidation by stimulating peroxisome proliferator-activated receptor *α* (PPAR*α*) [165]. Each of these mechanisms would be expected to reduce hepatic apolipoprotein B-100 synthesis and reduce VLDL synthesis and secretion. Indeed, a number of studies show that reductions in postprandial lipemia with n-3 fatty acids are associated with lower synthesis of apolipoprotein B-100 [160, 166–168]. A more recent study showed that n-3 fatty acids decreased postprandial apolipoprotein B-48 concentrations by 22% suggesting that the reduction in postprandial lipemia with n-3 fatty acids are also related to improvements in chylomicron metabolism [28].

### 5.1. n-3 Fatty Acids and Exercise

Potentially overlapping mechanisms have prompted some to explore the combined effects of n-3 fatty acids and aerobic exercise on postprandial lipemia. An early investigation by Thomas et al. [161] showed that a combination of short-term n-3 fatty acids (4 g/d) and a single bout of exercise failed to reduce postprandial lipemia in sedentary individuals. The authors suggested that the reduction in fasting TGs by n-3 fatty acids reduced the postprandial TG-lowering effects of exercise. In a follow-up study by the same group, aerobic exercise and n-3 fatty acids reduced postprandial lipemia in recreationally active individuals in an additive fashion [169]. It was hypothesized that trained and untrained individuals may respond differently to n-3 fatty acid and aerobic exercise interventions and that if sedentary individuals were to become trained, that they might benefit from the combination of exercise and n-3 supplementation. To test this hypothesis, the authors examined the effects of 4 weeks of training in previously sedentary overweight men and women [170]. n-3 fatty acids reduced fasting and postprandial TG concentrations, but the addition of exercise training produced no additional benefits. In a more recent study, n-3 fatty acid supplementation for 16 weeks with an exercise intervention (which did not produce weight loss) provided significantly greater reductions in TG AUC_I_ compared to n-3 fatty acids alone in viscerally obese individuals [158]. From the available literature, it is difficult to form a straightforward conclusion about the effects of combining n-3 fatty acids and exercise on postprandial lipemia. Future studies will be required to examine whether combination therapy is superior in hypertriglyceridemic versus normotriglyceridemic individuals and/or if the disparity in results are due to differences in the duration of treatment.

## 6. Quantifying Postprandial Lipemia

Assessment of postprandial lipemia has traditionally occurred over the course of 6–8 hours in laboratory settings. The extensive length of time and variety of macronutrient manipulations used has hindered the transition of oral fat tolerance tests to the clinic. Furthermore, the optimal mathematical representation for reporting postprandial lipemia with multiple time points continues to be debated. The traditional approach has been to report the absolute increase in TG concentrations that occur over the course of 6–8 hours with measurements conducted at 1-2 hour intervals. This technique is complemented by using a summary measurement referred to as the AUC which uses the trapezoidal rule [171] to calculate the average area under the absolute curve. While this strategy provides a valid assessment of the absolute response to the test meal, it does not factor in effects of interventions which influence baseline fasting TG concentrations. Since fasting TG concentrations are highly associated with the absolute hourly response and total TG AUC, it can be argued that absolute reductions in the postprandial response to a meal challenge are due to the reduction in fasting TGs as opposed to effects of the intervention during the postprandial period. To account for the effects of an intervention on fasting concentrations prior to a meal, the incremental AUC has been used as a method to control for reductions in fasting TG concentrations. The incremental AUC factors out the fasting TG concentration from the hourly responses and produces a curve that strictly reflects the success of the intervention on postprandial serum TG concentrations. However, it should be cautioned that one study reported that the reproducibility of the incremental TG AUC is relatively low when compared to the total TG AUC suggesting this as a limitation for incremental AUC [172].

While it seems clear that the most effective mathematical strategy at this point is to use both the total and incremental AUC, we still have not adequately dealt with the clinical utility of these tests as it relates to the length of time required to conduct the tests. It is well known that TG concentrations peak in most individuals within a 4-hour period [173, 174] (although it may occur sooner in women than men) and that the 4-hour peak is highly related to the total 8 h postprandial lipemia response [175], which can be used for accurate estimation of the postprandial response in healthy and at-risk individuals. Additional studies provide evidence that an abbreviated 4 h test is appropriate and is highly correlated with the results of full length tests [172, 175, 176] with the caveat that it may be a better predictor in healthy lean or obese populations but not in individuals with hypertriglyceridemia [176].

### 6.1. Other Markers of Postprandial Lipid/Lipoprotein Metabolism

While the effects of a lifestyle intervention on the postprandial response to a high fat or mixed meal is most often measured by the magnitude and duration of the TG response, these responses provide little information about the source of lipids (exogenous or endogenous). As described above, a primary objective of current research programs is to determine the mechanisms by which aerobic exercise and dietary interventions reduce postprandial lipemia. One of the earliest methods used to evaluate the source of TGs following a meal is retinyl palmitate [177]. Retinyl palmitate is the ester of retinol (vitamin A) and palmitate which is given in concert with a test meal. In the intestinal mucosa, retinyl palmitate is incorporated into the chylomicron core where it is thought to remain during triglyceride hydrolysis [35, 178–180]. This method assumes that the retinyl ester remains associated with apolipoprotein B-48 and does not exchange with other lipoproteins. Numerous challenges [181–184] to this assumption have been observed suggesting that retinyl palmitate may not be an ideal approach to evaluate the contribution of exogenous and endogenous sources of lipoproteins following a meal.

A more accurate approach to quantifying the source of TRLs may lie in the direct measurement of apolipoprotein B-48. Apolipoprotein B-48 has a molecular weight (MW) of 264 kDa and is approximately 48% the mass of apolipoprotein B-100 with a MW of 550 kDa [185]. In this technique, TRLs are separated from plasma by ultracentrifugation and the concentrations of apolipoprotein B-48 and B-100 are quantified by HPLC or densitometry [8, 35, 186]. The postprandial apolipoprotein concentrations are subsequently reported in relative units or considered as AUC responses in a similar fashion to that used for TGs.

More sophisticated approaches to apolipoprotein quantification and kinetics have been employed using stable isotopes to examine whether defects in secretion and/or catabolism of apolipoproteins are responsible for hypertriglyceridemia following a meal. For example, Wong and colleagues [28] used a bolus of d_3_-leucine (5 mg/kg BW) with a high-fat meal in obese and lean individuals to evaluate chylomicron metabolism. Using this approach, the authors were able to show that central obesity results in an overproduction and impaired catabolism of apolipoprotein B-48 containing lipoproteins. Additional studies have used stable isotopes to label test meal fatty acid content to differentiate between exogenous and endogenous TGs where [U-^13^C] palmitate (5 mg/kg FFM) was administered in a liquid test meal. Lipids were isolated using a heptane/isopropanol extraction procedure and analyzed by LC/MS [83].

## 7. Conclusions and Future Directions

Moderate-high intensity aerobic and resistance exercise produce consistent reductions in postprandial lipemia when performed 30 minutes to 20 hours prior to mixed or high-fat meals. Energy deficits created by moderate-intensity exercise appear to be responsible for a considerable proportion of the decrease in postprandial TGs with exercise. However, studies using resistance and HII exercise suggest that other factors may be involved. Future studies are needed to compare the effects of resistance exercise and HII in men and women. Furthermore, studies are required to determine the impact of weight loss (produced by energy restriction and/or increased energy expenditure) on the acute postprandial lipemia response to exercise. On a practical level, the minimum threshold of exercise energy expenditure to lower postprandial lipemia in sedentary and active populations is crucial. Additional studies using innovative approaches are also needed to more thoroughly evaluate the mechanisms by which exercise lowers postprandial TGs and if these mechanisms are different between obese and nonobese populations, sex, and race/ethnicity.

Reductions in dietary carbohydrate without reducing energy intake also decrease postprandial lipemia. Future studies will be needed to determine the mechanisms by which low-carbohydrate (glucose and/or fructose) diets work. Studies using deuterated water could be used to evaluate de novo lipogenesis between tissues.

## Abbreviations

TRLs: Triglyceride rich lipoproteins
TG: Triglyceride
LPL: Lipoprotein lipase
HTG: Hypertriglyceridemia
HOMA-IR: Homeostasis model assessment-insulin resistance.
